# ISWI Remodelling of Physiological Chromatin Fibres Acetylated at Lysine 16 of Histone H4

**DOI:** 10.1371/journal.pone.0088411

**Published:** 2014-02-06

**Authors:** Henrike Klinker, Felix Mueller-Planitz, Renliang Yang, Ignasi Forné, Chuan-Fa Liu, Lars Nordenskiöld, Peter B. Becker

**Affiliations:** 1 Department of Molecular Biology, Adolf Butenandt Institut, Ludwig-Maximilians-Universität München, Munich, Germany; 2 Center for Integrated Protein Science Munich, Munich, Germany; 3 School of Biological Sciences, Nanyang Technological University, Singapore, Singapore; 4 Protein Analysis Unit, Adolf Butenandt Institut, Ludwig-Maximilians-Universität München, Munich, Germany; National Cancer Institute, United States of America

## Abstract

ISWI is the catalytic subunit of several ATP-dependent chromatin remodelling factors that catalyse the sliding of nucleosomes along DNA and thereby endow chromatin with structural flexibility. Full activity of ISWI requires residues of a basic patch of amino acids in the N-terminal ‘tail’ of histone H4. Previous studies employing oligopeptides and mononucleosomes suggested that acetylation of the H4 tail at lysine 16 (H4K16) within the basic patch may inhibit the activity of ISWI. On the other hand, the acetylation of H4K16 is known to decompact chromatin fibres. Conceivably, decompaction may enhance the accessibility of nucleosomal DNA and the H4 tail for ISWI interactions. Such an effect can only be evaluated at the level of nucleosome arrays. We probed the influence of H4K16 acetylation on the ATPase and nucleosome sliding activity of *Drosophila* ISWI in the context of defined, *in vitro* reconstituted chromatin fibres with physiological nucleosome spacing and linker histone content. Contrary to widespread expectations, the acetylation did not inhibit ISWI activity, but rather stimulated ISWI remodelling under certain conditions. Therefore, the effect of H4K16 acetylation on ISWI remodelling depends on the precise nature of the substrate.

## Introduction

The nucleosomal organisation of genomic DNA constitutes a barrier to DNA binding factors. Therefore, nucleosome positions have to be tightly, yet dynamically controlled to enable the interaction of regulators of replication and transcription programmes with their cognate DNA binding sites. Of key importance in these processes are chromatin remodelling factors, a conserved class of enzymes that utilize the energy from ATP hydrolysis to reposition, evict, and assemble nucleosomes [1], [2]. ISWI, a prominent member of this class of ‘remodelling’ ATPases, is the catalytic subunit of several different chromatin remodelling complexes [3], [4]. All ISWI complexes investigated to date mobilize nucleosomes by repositioning histone octamers along DNA in a process termed ‘nucleosome sliding’ [5], [6]. Furthermore, some of them, such as the ACF-type complexes that consist minimally of the non-catalytic subunit Acf1 in addition to ISWI, assist nucleosome assembly and introduce a regular spacing into nucleosome arrays *in vitro* [7]–[12]. *In vivo*, ISWI complexes are involved in multiple essential nuclear processes, such as transcription regulation, DNA repair, and the maintenance of chromatin higher order structure [13], [14]. Still, how ISWI complexes are targeted and regulated and how their biochemical properties are translated into various biological outcomes remains largely elusive.

Since the ATPase ISWI is able to slide nucleosomes *in vitro* in absence of associated complex subunits, it serves as valuable model for mechanistic analyses. ISWI engages the nucleosome via its ATPase domain about two helical turns off the nucleosomal dyad [15]–[18]. At this site, the N-terminal tail domain of histone H4 (referred to as ‘H4 tail’ hereafter) emanates [19]. Notably, full activation of ISWI requires a basic patch of the H4 tail (amino acids 16–20), more specifically the residues R_17_H_18_R_19_ [16], [20]–[24].

Besides regulating ISWI activity, the H4 tail is critically involved in the folding of chromatin fibres. It strongly promotes fibre condensation, mainly by interacting with an acidic patch formed by histones H2A and H2B of nearby nucleosomes [25]. Notably, deletion of the tail as well as its acetylation causes considerable decompaction of chromatin at the level of intra- as well as inter-fibre interactions *in vitro* [26]–[29]. Especially acetylation of lysine 16 (H4K16ac) dramatically reduces the compaction capability of chromatin arrays even in presence of linker histones that have a strong chromatin condensation effect [30]–[33]. This *in vitro* finding is in accordance with *in vivo* data that found the H4K16ac mark to be enriched in open and accessible chromatin regions [34]–[36].

Given the importance of the H4 tail for ISWI activity, it is conceivable that posttranslational modifications, especially of residues within the basic patch, may modulate ISWI catalysis. Indeed, several observations suggest that H4 tail acetylation – in particular on lysine 16 – inhibits the activity of ISWI complexes. For example, ISW2, an ACF-related complex in *S. cerevisiae*, showed reduced activity if the H4 tail of substrate mononucleosomes was acetylated on all four lysines [37]. Although the inhibitory effect of the tetra-acetylation on the ATPase activity of ISW2 was minor (1.1-fold), acetylated mononucleosomes were repositioned 1.4-fold more slowly than unmodified ones. In a different study, site-specific acetylation of lysines 12 or 16 reduced the ATPase activity of *Drosophila* ISWI to approximately 65% when H4 tail peptides were used along with DNA to mimic nucleosome stimulation [23]. Peptide competition assays further confirmed inhibition of ISWI activity by these acetylation marks [38]. Moreover, H4K16ac markedly reduced *Drosophila* ACF-catalysed mononucleosome sliding by a factor of 2.7 [30]. Notably, contrary to the inhibitory effect of H4 tail acetylation observed in the context of mononucleosomes and tail peptides, hyperacetylation of chromatin fibres permitted faster remodelling by *Drosophila* ACF-type complexes and ISWI [39]. This might reflect better accessibility of the nucleosome and H4 tail in the acetylated, unfolded fibres.

Also studies in physiological settings hint at a complex interplay of H4K16ac and ISWI activity. Male *Drosophila* larvae lacking ISWI expression show striking decondensation of the X chromosome in spreads of polytene chromosomes [38], [40]. The male X chromosome is characterized by H4K16ac enrichment due to the activity of the dosage compensation machinery [36]. This activity was found to be necessary and sufficient for the X chromosome decompaction observed upon ISWI loss. Thus, it was proposed that ISWI complexes are involved in chromatin compaction by counteracting the decondensing effect of H4K16ac. In this model, reduced activity of ISWI on H4K16ac-carrying nucleosomes, as suggested by *in vitro* data, leads to an inherently more open structure of the male X chromosome.

However, the situation is more complex. Complete ISWI depletion is accompanied by striking loss of linker histone H1 from chromatin along with global chromatin decondensation [41]. This finding is in line with *in vitro* experiments showing that *Drosophila* ISWI and ACF can assist H1 incorporation into chromatin arrays [42] and slide H1-associated nucleosomes, although with reduced efficiency [43]. An ISWI complex may therefore contribute to H1 homeostasis in chromosomes. Nevertheless, the mechanism – direct or indirect – through which ISWI promotes chromatin condensation *in vivo* and the contributions of H4K16ac as well as H1 in this process remain unclear. Yet, investigating the interplay of these factors in cells is complicated and hampered by indirect effects. *In vitro* experiments on the other hand thus far mostly involved non-physiological substrates, like mononucleosomes or H4 tail peptides.

Here, in appreciation of the particular influence of H4K16ac on chromatin fibre folding, we investigated the effect of the modification on the remodelling activities of *Drosophila* ISWI and ACF in the context of fully defined, *in vitro* reconstituted chromatin arrays [44]. These arrays consisted of 25 nucleosomes that were either bound or unbound by linker histone and reflected the physiological chromatin substrate in several respects. They featured a nucleosomal repeat length of 197 bp, which is typically found in *D. melanogaster* [45]. H4K16ac was demonstrated earlier to enhance linker DNA accessibility [46] and to decrease salt-dependent compaction of similar arrays with different nucleosome spacing [30]–[32], whereas the linker histone strongly promoted condensation [31], [47]. The abundance of H1 in the nuclei of *Drosophila* cells suggests that the majority of nucleosomes is associated with the linker histone, forming so-called chromatosomes [48]. Therefore, chromatosome arrays are expected to resemble the physiological chromatin fibre even more accurately than nucleosome arrays. Employing the reconstituted nucleosome and chromatosome arrays as remodelling substrates, we found that in contrast to widespread expectations the homogenous acetylation of H4 on lysine 16 does not negatively affect ISWI and ACF activity.

## Results

### Reconstitution of nucleosome arrays carrying H4K16ac

To investigate the influence of H4K16ac on ISWI catalysis, we reconstituted 25-mer nucleosome arrays carrying either unmodified or site-specifically acetylated H4 from recombinant histones *in vitro*. The acetylated H4 was generated using a semi-synthetic approach employing native chemical ligation as illustrated in Figure S1A [32]. This method introduces a lysine analogue (K_S_) at position 20 of histone H4 (H4K_S_20). K_S_ is identical to lysine except for a thioether in its side chain (Figure S1A, B). Previous studies found H4K_S_20-carrying nucleosome arrays to behave similar to unmodified arrays in salt-dependent compaction [49]. Furthermore, remodelling by hACF was undisturbed in presence of trimethylated H4K_S_20 [50]. Tandem mass spectrometry confirmed quantitative acetylation of the semi-synthetic histone on lysine 16 (Figure S1C, D).

**Figure 1:**
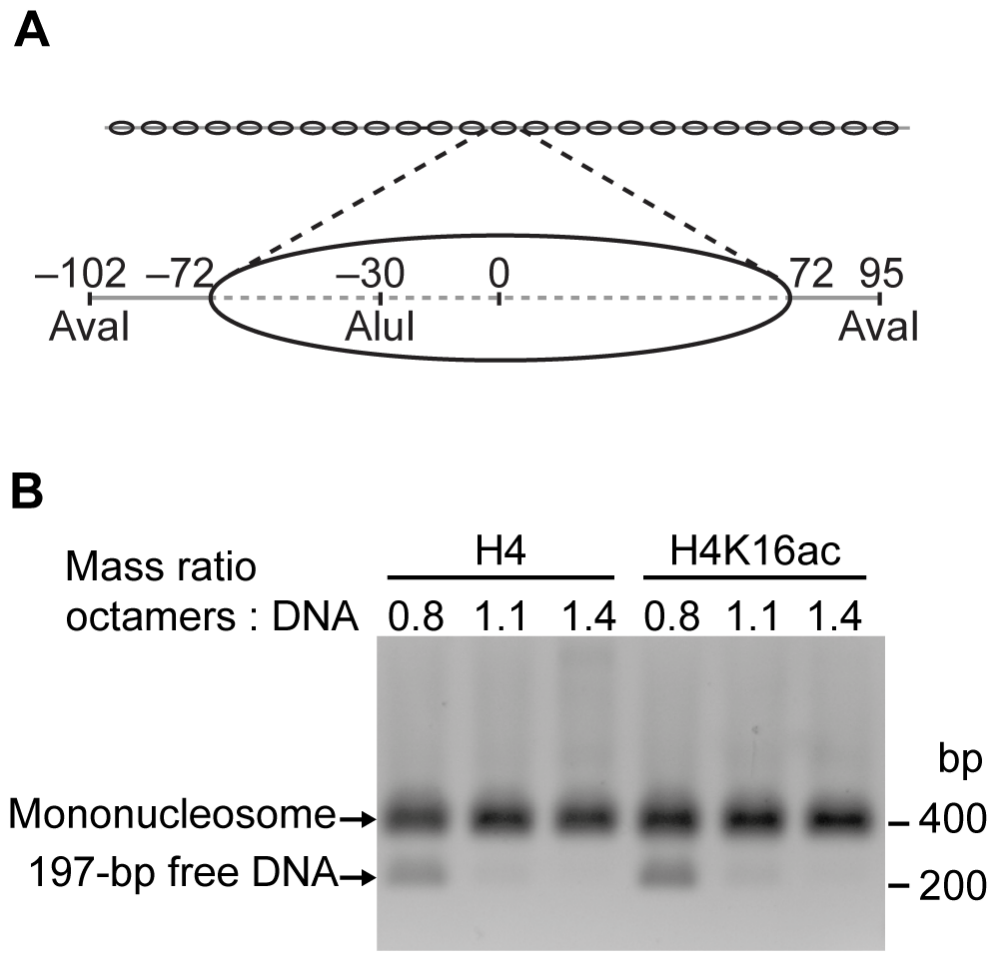
Nucleosome array reconstitution. (A) Schematic depiction of the nucleosome arrays (DNA: grey line; nucleosome positions: ovals). The array DNA comprised 25 repeats of a 197 bp fragment (magnification) harbouring the Widom-601 nucleosome positioning sequence (dashed line). Numbers indicate positions of restriction enzyme sites and nucleosome boundaries with respect to the nucleosomal dyad axis (0). (B) AvaI digests of purified nucleosome arrays reconstituted with increasing amounts of unmodified (H4) or acetylated (H4K16ac) octamers. The reactions were loaded onto a native agarose gel and DNA visualized by ethidium bromide stain. (bp: base pairs).

We assembled the acetylated H4 into histone octamers and reconstituted regularly spaced nucleosome arrays applying a method first described by the Rhodes lab [44]. To yield a regular spacing of nucleosomes, we assembled the arrays by salt gradient dialysis on a linear DNA fragment comprising 25 repeats of a 197 bp derivative of the Widom-601 nucleosome positioning sequence (Figure 1A) [51], [52]. To prevent oversaturation of the arrays with histones during assembly, short DNA fragments were present during the reconstitution. These DNA fragments did not harbour nucleosome positioning sequences and served as low affinity competitors that bound excess histones.

Through titrations, we determined how much of the octamers was needed to saturate the DNA template. After assembly, the nucleosome arrays were purified by MgCl_2_ precipitation, which removed histone-bearing and free competitor DNA fragments [53]. Saturation was controlled on native agarose gels (Figure S2B), and histone stoichiometry was assessed on Coomassie-stained SDS gels (Figure S2A). We independently confirmed saturation by digesting the arrays with the restriction enzyme AvaI that cuts in the linker DNA between Widom-601 repeats (Figure 1). On native gels, mononucleosomes migrated more slowly than the corresponding free DNA, and disappearance of the 197 bp DNA band with increasing histone octamer concentrations indicated full occupancy of the Widom-601 sites with nucleosomes. In a complementary approach, arrays were digested with AluI. In contrast to AvaI, the AluI site is located within the Widom-601 sequence and was occluded upon nucleosome formation (Figure 1A). None of the AluI sites were cleaved when the arrays were saturated with octamers (Figure S2C). All quality controls indicated that comparable amounts of acetylated and unmodified histone octamers were required to reach saturation (Figures 1B; S2B, C). We subjected each array preparation to the described quality controls to assure full nucleosome occupancy.

### H4K16ac does not influence ISWI ATPase activity

**Figure 2:**
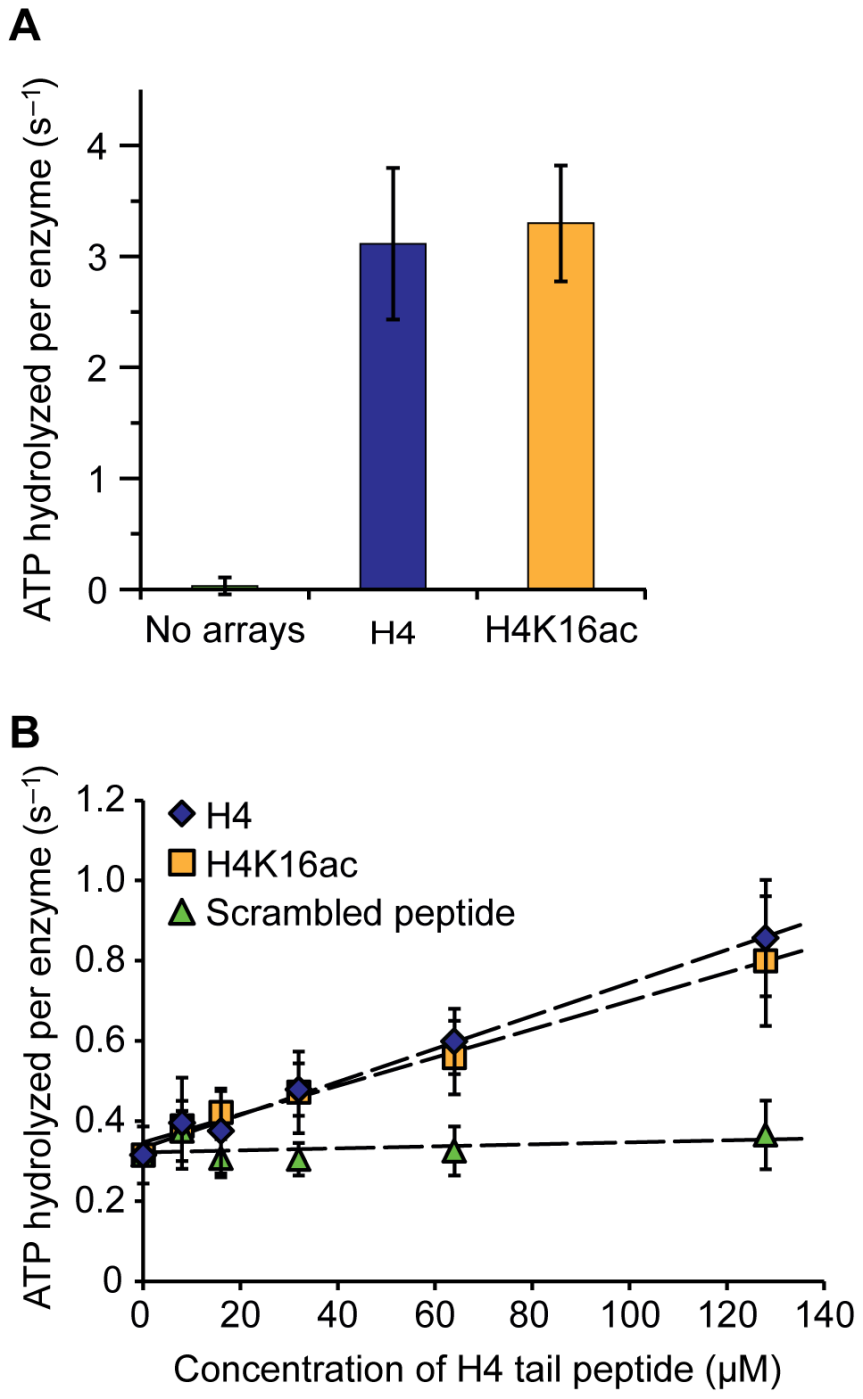
ISWI ATPase activity is not influenced by H4K16ac. (A) Steady-state ATPase assay. ATPase activity of ISWI (100 nM) was stimulated with saturating concentrations of nucleosome arrays (600 nM) carrying unmodified (H4) or acetylated H4 (H4K16ac). ATP hydrolysis rates in presence of saturating concentrations of ATP (1 mM) were determined. Control reactions did not contain nucleosome arrays. Error bars represent standard deviations (No arrays: n = 4; H4 and H4K16ac: n = 5). (B) ISWI (350 nM) was stimulated with DNA (1.2 mg/ml salmon sperm DNA) and increasing concentrations of an unmodified or H4K16ac-carrying histone H4 N-terminal peptide (H4 tail peptide). ATP hydrolysis rates were determined as above at 1 mM ATP. A peptide with scrambled amino acid sequence of the H4 tail harbouring an acetylated lysine residue served as control. Data were fit to lines to extract slopes (dashed lines; H4: 4.1*10^3^ s^−1^ M^−1^; H4K16ac: 3.5*10^3^ s^−1^ M^−1^). Error bars display standard deviations (n = 3–4).

Previous studies suggested that acetylation of H4K16 on mononucleosomes or H4 tail peptides in conjunction with DNA reduces ISWI and ACF activity [23], [30], [37], [38], [54]. Our aim was to test whether H4K16ac influences ISWI activity also in the context of nucleosome arrays, a more physiological substrate. Therefore, we saturated ISWI with unmodified or acetylated nucleosome arrays and measured ATP turnover at steady-state (Figure 2A). We controlled for saturation of ISWI with arrays by titrating the nucleosomal substrate (Figure S3). Surprisingly, ATP turnover increased by the same amount with both array types. Thus, at the level of nucleosome arrays H4K16ac did not influence ISWI ATPase activity.

On the basis of previous studies, we also tested the stimulation of the ATPase activity of DNA-bound ISWI by H4 tail peptides [23], [52], [54]. Notably, also in this assay H4K16ac did not affect ATP turnover by ISWI (Figure 2B). Acetylated H4 peptides stimulated only marginally (1.2-fold) worse than unmodified ones, as can be estimated from the slopes of the curves. This 1.2-fold effect is slightly smaller than the previously documented one of ∼1.5-fold in similar experiments [23], [54] and well within the error of our assay. We conclude that, contrary to common interpretations of published data, the acetylation of H4K16 does not significantly influence the ATPase activity of ISWI under our assay conditions.

### H4K16ac does not inhibit remodelling by ISWI and ACF

**Figure 3:**
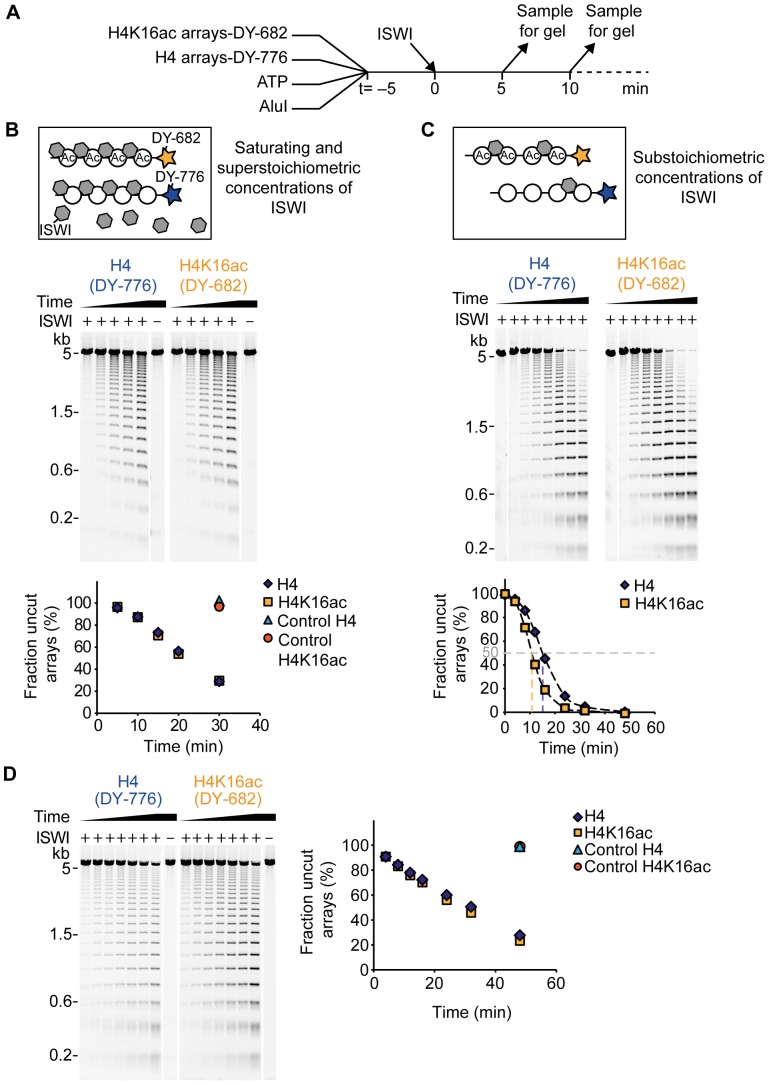
ISWI and ACF remodelling activity is not inhibited by H4K16ac. (A) Scheme of the remodelling assay. Acetylated (H4K16ac) and unmodified (H4) arrays were labelled at one DNA end with the fluorescent dyes DY-682 and DY-776, respectively. Remodelling reactions contained both array types along with ATP, AluI and the remodeller. Samples of the reaction were taken at different time points (t) and the DNA fragments were analysed on an agarose gel. (B) Top: Schematic depiction of the reaction conditions of the remodelling assay. (Ac: acetylated arrays). Middle: Exemplary result of a remodelling time course with ISWI (500 nM). Nucleosome concentration was 25 nM per array type and ATP concentration was 1 µM. All samples were run on the same agarose gel and the two fluorescent labels were visualized separately by scanning the gel at the respective wave lengths. Lanes were rearranged for presentation purposes. Control reactions did not contain ISWI (–). Bottom: Quantification of remodelling progress. Based on the fluorescent signal intensity the fraction of uncut array DNA was determined for each gel lane and plotted against the remodelling time. (C) Remodelling assay as in B, but with ISWI and nucleosome concentrations of 5 nM and 100 nM, respectively. ATP concentration was 200 µM. In the plot shown in the bottom panel, the remodelling times needed to reach 50% cut array DNA were interpolated by connecting the data points by smooth lines (Excel; Microsoft). (D) Exemplary result of a remodelling time course with ACF. Reaction conditions were as in C. (kb: kilobases).

H4K16ac may influence ISWI remodelling activity even though ATP hydrolysis remained unaffected. Using the 25-mer nucleosome arrays as substrates, we followed the remodelling activity of ISWI by monitoring accessibility of the AluI restriction site [43], [52]. This site was protected from cleavage by a positioned nucleosome at the outset of the reaction (Figure 1A), but was rendered accessible upon remodelling. Therefore, the array DNA got fragmented over time. To be able to directly compare remodelling of unmodified and acetylated arrays in the same reaction, we differentially labelled both array types with a unique fluorescent tag at one DNA end. Figure 3A illustrates the set-up of the remodelling assay.

To discriminate the influence of the acetylation on different steps of ISWI catalysis, we performed the assay under two conditions. First, we saturated the arrays with ISWI by adding an excess of enzyme at high concentrations (Figure 3B). In this experimental setting, remodelling velocity is expected to depend only on catalytic steps after substrate binding, as all ISWI binding sites on the arrays were occupied regardless of affinity (Figure 3B top panel). Under these conditions, H4K16ac-carrying arrays were remodelled with the same velocity as unmodified arrays (Figure 3B middle and bottom panel). Thus, we conclude that the acetylation did not affect catalytic steps subsequent to nucleosome binding.

Yet, it remained possible that H4K16ac changed the affinity of ISWI for the nucleosomes. To investigate this possibility, we added ISWI in substoichiometric concentrations to the nucleosome arrays. Under these conditions, ISWI distributed among the unmodified and acetylated arrays according to its affinity (Figure 3C top panel). In this setting, acetylated arrays were remodelled modestly faster than unmodified ones (Figure 3C middle and bottom panel). To quantify the difference, we interpolated the time needed to cut 50% of the arrays (dashed lines in Figure 3C bottom panel; H4: 15 min; H4K16ac: 11 min). These values differed by a factor of 1.4. Several independent repetitions of the experiment with varying enzyme and array concentrations confirmed these results (data not shown). As long as ISWI was substoichiometric to nucleosomes, unmodified arrays were reproducibly remodelled 1.3–1.7-fold more slowly than acetylated arrays (1.5-fold on average). Since we already showed that ISWI remodelling subsequent to substrate binding was unaffected by H4K16ac (Figure 3B), the difference in remodelling velocity observed here hints at a preferred binding of ISWI to acetylated arrays. Our results contrast previous studies reporting reduced remodelling activity of ACF-type complexes in presence of H4K16ac at the level of mononucleosomes [30], [37], but reiterate observations of enhanced remodelling activity on hyperacetylated nucleosome arrays [39].

To test whether the discrepancy between our and published data was due to working with the isolated ISWI enzyme as opposed to the ACF complex, we repeated the remodelling assay with ACF. To capture possible effects of the acetylation on binding affinity as well as later catalytic steps, we employed substoichiometric concentrations of ACF. We found ACF to remodel H4K16ac-carrying and unmodified arrays with comparable velocities (Figure 3D). Thus, we reason that neither ACF binding affinity nor further steps of the remodelling mechanism were influenced by the acetylation.

In principle, the discrepancy between our and published findings could be accounted for by differences in the design of the employed remodelling assays. In the remodelling assay used here, accessibility of AluI sites could be caused by different remodelling events. The sites could, for example, be exposed by sliding a nucleosome away from its original position, by nucleosome eviction, or by transient changes in the canonical nucleosome structure. Previous studies reporting reduced remodelling of ISWI and its complexes in presence of H4K16ac exclusively looked at sliding events [30], [37]. Therefore, we next performed sliding assays.

**Figure 4:**
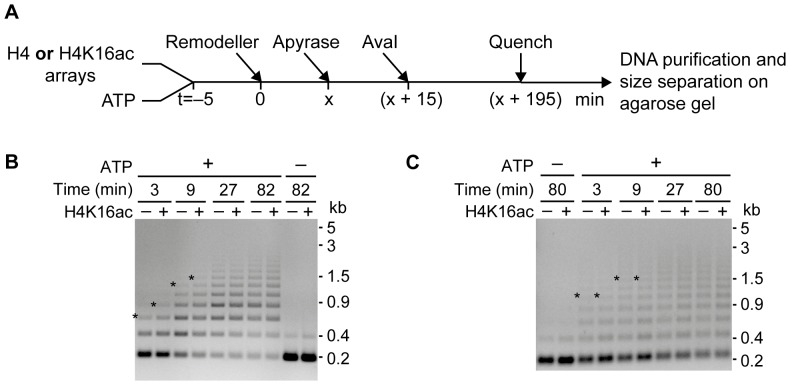
Sliding activity of ISWI and ACF is not reduced by H4K16ac. (A) Schematic depiction of the sliding assay. Each reaction contained either unmodified (H4) or acetylated (H4K16ac) arrays (30 nM) along with ATP (125 µM) and was started by addition of the remodeller (5 nM). At different time points (x) samples were taken, remodelling was quenched by ATP depletion with apyrase, and AvaI was added. AvaI activity was quenched with EDTA (40 mM) and SDS (0.4%) before purifying the DNA fragments and size-separating them on an agarose gel. (B) and (C) ISWI and ACF sliding time courses, respectively. The asterisks mark the slowest migrating still well visible DNA band in the respective gel lanes. In control reactions ATP was depleted before remodeller addition (– ATP). (kb: kilobases).

To follow nucleosome sliding, remodelling-dependent changes in the accessibility of the AvaI sites located in the linker DNA between nucleosomes (Figure 1A) were monitored [52], [55]. Protection of the initially exposed AvaI sites was indicative of nucleosome sliding (Figure 4A). We found that ISWI repositioned acetylated nucleosomes slightly faster than unmodified ones, as evidenced by the accumulation of longer DNA fragments at earlier time points (Figure 4B). At equilibrium, the DNA patterns were comparable for both array types (Figure 4B, time points 27 and 82 min). Contrary to ISWI, ACF repositioned unmodified and acetylated nucleosomes with equal velocity (Figure 4C). These observations closely reiterate the results we obtained with the remodelling assay under similar conditions (Figure 3C, D).

Taken together, the acetylation neither inhibited ISWI nor ACF remodelling at the level of nucleosome arrays. Rather, ISWI showed a modest preference for remodelling H4K16ac-carrying arrays.

### Reconstitution of chromatosome arrays carrying H4K16ac

**Figure 5:**
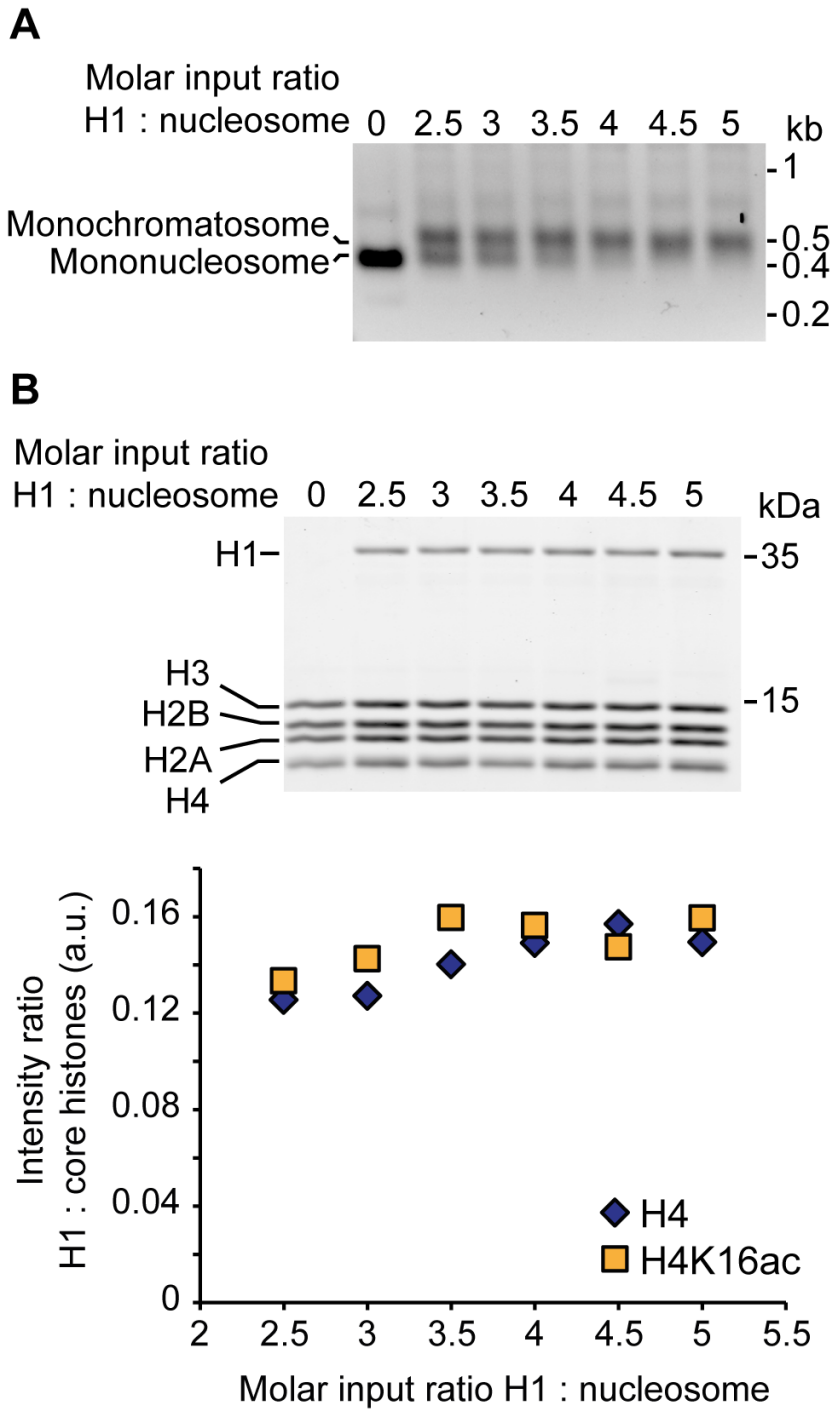
Reconstitution of chromatosome arrays. (A) AvaI digests of chromatosome arrays carrying H4K16ac. Chromatosome arrays were reconstituted with increasing amounts of H1, purified by MgCl_2_ precipitation and digested to monomers with AvaI. Mononucleosomes were separated from chromatosomes on native agarose gels and visualized by ethidium bromide stain. Faint additional bands may contain coprecipitating competitor DNA or multimers arising from incomplete AvaI digest. (kb: kilobases). (B) Analysis of the relative ratio of H1 to core histones in chromatosome arrays. Top: Chromatosome arrays from the H1 titration shown in A were loaded onto SDS gels and the protein content visualized by Coomassie stain. The gel of the acetylated chromatosome arrays is depicted. Bottom: Quantification of the relative ratio of H1 to core histones. The theoretically expected ratio of 0.24 for a 1:1 stoichiometry of H1 to octamers was not reached, presumably because the Coomassie staining did not linearly correlate with the molecular weight of the proteins [79]. (kDa: kilodaltons; a.u.: arbitrary units).

To investigate the effect of H4K16ac on the activity of ISWI on a substrate that resembles the *in vivo* situation even better than nucleosome arrays, we assembled arrays containing linker histone H1, so-called chromatosome arrays [44]. H1 was titrated to determine the ratio of H1 to nucleosomes needed in the reconstitution reactions to saturate the arrays. Saturation with H1 was tested by two complementary approaches. First, arrays were digested with AvaI into monomers. On native gels, monochromatosomes showed altered mobility in comparison to mononucleosomes, and disappearance of the mononucleosome band indicated quantitative formation of chromatosomes (Figure 5A for H4K16ac-carrying chromatosome arrays; data for unmodified arrays not shown).

Second, we controlled the relative stoichiometry of linker histone incorporation on Coomassie-stained SDS gels (Figure 5B). Increasing the H1 amounts up to a molar ratio of approximately four H1 per nucleosome in the reconstitution reactions led to a corresponding increase in H1 incorporation. At this point, a plateau in linker histone incorporation was reached, indicating saturation of primary binding sites in agreement with the results obtained in the AvaI digests (Figure 5A). Despite the buffering effect of competitor DNA, addition of H1 beyond the saturation plateau led to aggregation and loss of material during assembly (data not shown) [44].

Similar H1 amounts saturated unmodified and H4K16ac-carrying arrays (Figure 5B). Yet, unmodified chromatosome arrays tended to aggregate at lower H1 input amounts than acetylated ones (data not shown). The described quality controls were performed for every chromatosome array preparation to ensure saturation.

### H1 inhibits ISWI activity irrespective of H4K16ac

**Figure 6:**
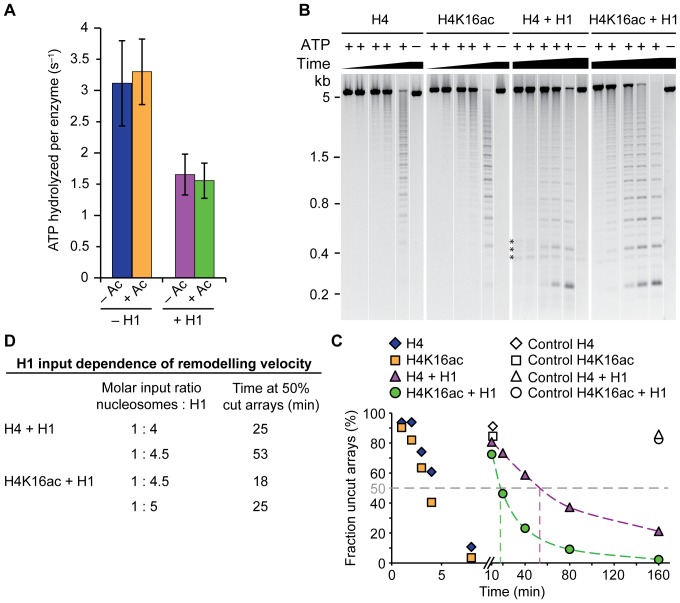
H1 inhibits ISWI activity. (A) Steady-state ATPase assay. Saturating concentrations (600 nM) of unmodified (– Ac) or acetylated (+ Ac) chromatosome arrays (+ H1) were employed to stimulate ISWI (100 nM) in presence of saturating ATP (1 mM). ATP hydrolysis rates for nucleosome arrays (– H1) were taken from Figure 2. Error bars represent standard deviations (– H1: n = 5; + H1 n = 6). (B) Exemplary remodelling assay using nucleosome and chromatosome arrays (200 nM) as substrates for ISWI (50 nM) at 100 µM ATP. The chromatosome arrays had been reconstituted with a 1:4.5 molar ratio of nucleosomes to H1. The assay was performed as in Figure 3 except that the arrays lacked a fluorescent label and all array types were tested in separate reactions. The DNA was visualized by ethidium bromide stain. All samples of a reaction were loaded onto one gel and empty lanes were spliced out. ATP was omitted from control reactions (–). The most prominent DNA bands comprise multiples of 197 bp reflecting the distance between two AluI sites within the array (Figure 1A). Faint interspersed bands arose from a single AluI cut; they harbour one of the ends of the array DNA and therefore deviate in length from the internal cleavage products. Asterisks mark DNA bands originating from contaminating competitor DNA. (kb: kilobases). (C) Quantification of the experiment depicted in B. Analysis was done as in Figure 3C. (D) Summary of the remodelling activity of ISWI on chromatosome arrays reconstituted with different input amounts of H1 (see B and Figure S4).

We first probed the effect of H1 on the steady-state ATP hydrolysis of ISWI. Under saturating array concentrations, the presence of H1 reduced ISWI ATP turnover by a factor of two and acetylation did not cause additional effects (Figure 6A).

We next tested ISWI remodelling activity on chromatosome arrays. In contrast to the remodelling assay described in Figure 3, we performed the assay with unlabelled arrays and set up separate reactions for each array type. H1 incorporation markedly reduced remodelling velocity of ISWI, confirming published results (Figure 6B, C) [43]. However, we hesitated to quantify the effect of the linker histone on ISWI remodelling because the reduction in AluI accessibility on chromatosomes might in part be caused by remodelling-independent, inherent properties of the arrays. H1 is expected to occlude about 20 bp of the 50 bp long linker DNA [56]. This reduces the probability to expose an AluI site simply due to restricted sliding possibilities.

Nevertheless, we could use the assay to quantitatively compare the remodelling activity of ISWI on chromatosome arrays harbouring unmodified and acetylated H4. H4K16ac seemed to counteract the inhibitory effect of H1 as indicated by faster remodelling of acetylated arrays (Figure 6B, C). This observation was reproducible for different array preparations when comparing ISWI activity on pairs of arrays reconstituted with the same ratio of H1 to nucleosomes in the assembly reactions (data not shown).

However, control experiments showed that remodelling was markedly dependent on the H1 to nucleosome ratio present in the reconstitution reactions. Although all arrays were saturated with H1 according to our quality controls, they were remodelled with different velocity (Figure 6D). Increasing the amount of H1 in the array assembly reactions by as little as 10% reduced the remodelling velocity considerably. Accordingly, using slightly more H1 in the reconstitution reactions of acetylated in comparison to unmodified arrays resulted in equal ISWI remodelling activity on both array types (Figure 6D). Therefore, we cannot exclude that the observed faster remodelling of H4K16ac-carrying chromatosome arrays at equal H1 input amounts simply reflected subtle differences in H1 incorporation. The sensitivity of ISWI towards minor differences in H1 saturation detected in the remodelling assay was not apparent in the ATPase activity of the enzyme (data not shown).

In summary, linker histone H1 reduced the ATPase activity of ISWI by two-fold irrespective of the acetylation status of H4K16. However, the strong dependency of ISWI remodelling on slight variation of linker histone input during reconstitution together with the inherent difficulties of working with the sticky H1 protein limited our ability to interpret the tendency of H4K16ac to ameliorate the inhibition of remodelling of chromatosome arrays.

## Discussion

### ISWI regulation by H4K16ac

It is firmly established that the H4 tail plays a crucial role in the nucleosome sliding mechanism of ISWI [5], [6]. A recent study provided mechanistic insights by identifying a peptide motif in the N-terminus of ISWI (AutoN) that resembles the basic patch of histone H4 [54]. This motif was suggested to interact with the ATPase domain and autoinhibit ISWI in absence of a nucleosome substrate. Yet, the study proposed also subsequent steps of ISWI catalysis to be promoted by the H4 tail, and these might involve other motifs than the basic patch.

To date, it is still unclear how modifications of the H4 tail affect nucleosome remodelling. Most previous studies suggested that – at the level of single nucleosomes – acetylation of H4K16 within the basic patch inhibits ISWI activity, but the extent and nature of the effect remained controversial [23], [30], [37]–[39], [54], [57]. We reassessed the effect of H4K16ac on ISWI activity in the context of defined, folded nucleosome and chromatosome arrays that resemble the physiological remodelling substrate. In our quantitative biochemical analyses, the acetylation neither inhibited ISWI nor ACF activity.

In contrast to the prevalent interpretation of earlier reports [23], [37], [54], we found the maximal ATP turnover by ISWI undisturbed by H4K16ac in the context of chromatin arrays. Small effects of the acetylation observed upon stimulation of DNA-bound ISWI with H4 peptides were well within the experimental variability of our carefully controlled assays and therefore should not be interpreted any further.

Not only the ATPase, but also the remodelling activity of ISWI on nucleosome arrays was not inhibited by H4K16ac. Differential labelling of unmodified and acetylated arrays allowed us to analyse remodelling of both substrates in the same reaction. Therefore, the assay was very sensitive to H4K16ac-dependent variations in remodelling progress, and ISWI activity was easily quantifiable. Tuning the assay conditions by varying the enzyme or substrate concentrations permitted to distinguish effects of the acetylation on different steps of ISWI catalysis. Due to the competitive substrate conditions, relative affinities of ISWI to the two array types could be isolated. Whereas H4K16ac did not influence steps of ISWI remodelling subsequent to nucleosome binding, our results indicated preferred binding to the acetylated fibre. This preference could simply reflect a higher affinity of ISWI for nucleosomes carrying H4K16ac. Alternatively, H4 tail availability and access of ISWI to the nucleosome substrate might be facilitated by H4K16ac-promoted unfolding of the fibre [30]–[32], [39], [46]. Other scenarios are also possible. For example, H4K16 acetylation may facilitate dimerization of ISWI [18]. In any case, the observed difference in remodelling velocity was modest (1.5-fold) and absent when ISWI was part of the physiologically relevant ACF complex.

### Regulation of ISWI by the linker histone H1

ISWI can remodel chromatosomes in the context of arrays, although with reduced efficiency in comparison to nucleosomes [43]. Here, we confirmed this finding and additionally report a two-fold reduction in ISWI ATPase activity in presence of the linker histone. This reduction was independent of H4K16 acetylation.

The nature of the inhibitory effect of H1 on ISWI activity remains to be determined. It is conceivable that a combination of mechanisms is in play. For example, H1 might directly hinder nucleosome sliding by blocking the entry or exit of DNA [58]–[60]. Association of H1 with the nucleosome is furthermore expected to interfere with productive ISWI interaction via the ISWI SANT-SLIDE domain, which is required for efficient nucleosome sliding [5], [6]. Moreover, H1 binding effectively reduces the accessible length of linker DNA, which might lead to decreased ISWI activity, as ISWI as well as ACF activity depends on the linker length [16], [61]–[65]. Finally, the high degree of array compaction induced by H1 incorporation [31], [47] might hinder ISWI access and chromatosome repositioning. The latter effect may be partially antagonized by the chromatin unfolding effect of H4K16ac [31], resulting in enhanced remodelling of acetylated chromatosome arrays. Although an effect of H4K16ac was not apparent in the ATPase assay, it remains possible that H4K16ac facilitates remodelling of chromatosome arrays.

We were surprised about the pronounced dependency of ISWI remodelling velocity on small variations in the amount of linker histone during array reconstitution and suspect that excess H1 readily occupies secondary binding sites in fibres [66]–[68], thereby affecting ISWI remodelling. Unfortunately, we cannot exclude subtle differences in H1 stoichiometry between acetylated and unmodified chromatosome arrays, which hampered analysing the effect of the H4K16 acetylation on ISWI remodelling.

Investigating remodelling on chromatin fibres with physiological nucleosome spacing and linker histone content allowed us to shed light on the effect of H4K16 acetylation on ISWI catalysis. Our study highlights the usefulness of the *in vitro* system for the dissection of the function of histone modifications. A technical challenge that needs to be tackled is to generate histones with combinatorial histone marks, as they are emerging to be of key importance in regulating chromatin processes [69], [70].

### *In vivo* implications

ISWI activity *in vivo* has been suggested to promote chromatin compaction [13], [41], [71], whereas H4K16ac seems to be involved in the establishment and maintenance of decondensed regions of chromatin [34]–[36]. Common models postulate that H4K16ac contributes to chromatin decompaction, amongst others, by inhibiting ISWI [1], [38]. Notably, this mechanism was proposed to play a role in dosage compensation in *Drosophila*. Our findings do not support the simplest of such models, as we did not observe reduced activity of ISWI in presence of the acetylation. However, *in vivo* the local context of the chromatin fibre is expected to influence ISWI activity at regions enriched in H4K16ac. This includes associated factors, histone variants and modifications, as well as the concentration of remodelling complexes, their subunit stoichiometry and modification state.

## Materials and Methods

### Expression and purification of remodelling enzymes

#### ISWI

*D. melanogaster* ISWI harbouring an N-terminal His_6_-TEV tag was bacterially expressed (BL21(DE3)) and purified as described [72]. In brief, a nickel affinity purification was performed by FPLC using a HisTrap column (GE healthcare) in 50 mM Tris-Cl pH 7.4, 300 mM NaCl and 20–400 mM imidazole. TEV-cleavage was followed by another nickel affinity chromatography step to remove uncleaved protein and His-tagged TEV protease. The flow-through was applied to a Mono S column (GE Healthcare) in 15 mM Tris-Cl pH 8, 1 mM β-mercaptoethanol and 100–2000 mM NaCl. For the final gel filtration, a Superdex 200 column (GE healthcare) in 50 mM Hepes-KOH pH 7.6, 0.2 mM EDTA, 200 mM KOAc, 10 mM β-mercaptoethanol was used. Enzyme concentration was determined by absorption measurement at 280 nm (extinction coefficient: 119950 cm^−1^ M^−1^). The pPROEX-HTb-based expression plasmid was a kind gift from C. Mueller (EMBL, Heidelberg).

#### ACF

*D. melanogaster* ACF complex was purified from Sf21 cells co-expressing flag-ISWI and Acf1-flag from baculovirus constructs. The baculovirus stocks were kind gifts from C. Wu [73] and J. Kadonaga [74], respectively. Virus-infected Sf21 cells were harvested, resuspended in 100 mM Tris-Cl pH 7.8, 500 mM KOAc, 10% glycerol supplemented with protease inhibitors, and lysed by ultrasonication (Branson). Flag affinity purification was performed using M2 agarose beads (Sigma). Contaminations as well as excess flag-ISWI were removed by Mono Q ion exchange chromatography (12 mM Tris-Cl pH 8, 1 M urea, 1 mM DTT, and 240–880 mM NaCl) followed by Superose 6 gel filtration (100 mM Tris-Cl pH 7.8, 500 mM KOAc, 1.5 mM Mg(OAc)_2_, 1 M urea, 10% glycerol, 10 mM DTT, 0.2% CHAPSO) (both columns GE Healthcare). Fractions containing monomeric ACF were pooled, concentrated (Microcon-30 kDa Centrifugal Filters; Millipore) and flash frozen in liquid nitrogen. Concentration of the complex was determined by measuring absorption at 280 nm (extinction coefficient: 244220 cm^−1^ M^−1^).

### Reconstitution of nucleosome and chromatosome arrays

#### DNA preparation

The array DNA, comprising 25 consecutive repeats of a 197 bp Widom-601 nucleosome positioning sequence derivative, was excised with HincII and EcoRI from a pUC18-based plasmid (a kind gift from D. Rhodes, NTU, Singapore). The vector backbone was fragmented further to pieces that served as competitor DNA in the reconstitution reactions by either DraI or combinations of DraI with AseI and DdeI (all from NEB). For the assembly of chromatosome arrays, the vector backbone was cleaved with all three enzymes, yielding fragments not exceeding 445 bp, because in presence of H1 longer histone-bound competitor DNA fragments tended to co-precipitate with the arrays in the MgCl_2_ precipitation step. After restriction enzyme digest, the DNA was purified by phenol/chloroform/isoamyl alcohol extraction. For the fluorescent labelling, array DNA was purified from the vector backbone fragments (generated with DraI and DdeI) by PEG6000 (5.5–6%) precipitation. The EcoRI end of the array DNA was labelled with either dUTP-DY-682 or dUTP-DY-776 (Dyomics) using Klenow-exo^−^ polymerase (NEB). Unincorporated nucleotides as well as proteins were removed by phenol/chloroform/isoamyl alcohol extraction and purification over gel filtration-columns (Micro Bio-Spin P-30 Gel Column; Bio-Rad).

#### Generation of acetylated histone H4

*X. laevis* histone H4 quantitatively acetylated at lysine 16 (K16) was prepared as described previously [32] (see also Figure S1). Quantitative acetylation of K16 was controlled by tandem mass spectrometry analysis of histone H4 incorporated into arrays. The proteins of the arrays were loaded on an 18% SDS gel and stained with Coomassie. The H4 band was excised, chemically acetylated with deuterated acetic anhydride and digested into peptides using trypsin as described previously [75]. Tryptic peptides were injected in an Ultimate HPLC system (LC Packings Dionex). Samples were desalted on-line in a C18 microcolumn (300 µm i.d. ×5 mm, packed with C18 PepMap™, 5 µm, 100 Å; LC Packings) and peptides were separated with a gradient from 5 to 60% acetonitrile in 0.1% formic acid over 40 min at 300 nl/min on a C18 analytical column (75 µm i.d. ×15 cm, packed in-house with Reprosil Pur C18 AQ 2.4 µm; Doctor Maisch). The effluent from the HPLC was directly electrosprayed into a linear trap quadrupole-Orbitrap mass spectrometer (Thermo Fisher Scientific). The MS instrument was programmed to acquire survey full-scan MS spectra (m/z 718–730) in the Orbitrap with resolution R = 15,000 at m/z 400 (after accumulation to a “target value” of 500,000 in the linear ion trap) followed by the isolation to a target value of 10,000 and fragmentation by collision-induced dissociation of the masses corresponding to the second isotope of the one, two and three times acetylated H4 4–17 peptide (724.95, 723.44, 721.92 m/z). Typical MS conditions were spray voltage, 1.5 kV; no sheath and auxiliary gas flow; heated capillary temperature, 200°C; normalized collision-induced dissociation energy 35%; activation q = 0.25; and activation time  = 30 ms.

#### Octamer assembly

Histone octamers were reconstituted from bacterially expressed histones H2A, H2B, H3 and H4 that was acetylated (see above) or unmodified. Reconstitution was done as described [76] with the following modifications. Lyophilized histones were resolved in 20 mM Tris-Cl pH 7.5, 7 M guanidinium-HCl, 10 mM DTT. Dissolved histones were mixed and extensively dialysed against 10 mM Tris-Cl pH 7.5, 2 M NaCl, 1 mM EDTA pH 8, 5 mM β-mercaptoethanol. Assembled octamers were purified by gel filtration in the same buffer (Superdex 200), and stoichiometric incorporation of the histones was controlled by SDS-PAGE and Coomassie stain. The octamers were concentrated (Amicon Ultra-4 Centrifugal Filter Units 30 kDa; Millipore), flash frozen in liquid nitrogen, and stored at -80°C. Concentrations of the octamer preparations were determined by absorption measurement at 280 nm (extinction coefficient: 44700 cm^−1^ M^−1^). All histones comprised the *D. melanogaster* amino acid sequence, except for histone H4 that harboured the *X. laevis* sequence. *X. laevis* and *D. melanogaster* histone H4 vary in only one amino acid at position 1. This amino acid was shown not to be essential for ISWI activity in a previous study [23]. Furthermore, both *D. melanogaster* and *X. laevis* nucleosomes stimulate ISWI ATP hydrolysis [20], [23].

#### Linker histone H1

Native linker histone was purified from 0–12 h after egg laying *D. melanogaster* embryos as previously decribed [77] with the following modifications. The H1-containing supernatant of the second ammonium sulphate precipitation was subjected to phenyl sepharose chromatography (column volume: 20 ml; Phenyl Sepharose 6 Fast Flow; GE Healthcare). The pooled H1-containing fractions were subjected to extensive dialysis against 25 mM Hepes-KOH pH 7.6, 100 mM KCl, 0.1 mM EDTA, 10% glycerol, 5 mM β-mercaptoethanol. For the final cation exchange step, a Mono S column was used. H1-containing fractions were pooled, concentrated (Amicon Ultra-4 Centrifugal Filter Units 10 kDa; Millipore), and glycerol was added to 50% for storage at −20°C. H1 concentration was determined by Coomassie staining of SDS gels taking BSA as a reference. Band intensities were quantified using the Odyssey Infrared Imaging System (LI-COR). The yield from 60 g of embryos was ∼1 mg of H1.

#### Array reconstitution

Nucleosome as well as chromatosome arrays were reconstituted by salt gradient dialysis over approximately 24 h at 4°C [44]. The final buffer contained 10 mM Tris-Cl pH 7.6, 50 mM NaCl, 1 mM EDTA pH 8, 0.01% NP-40, 1 mM DTT. pUC18 vector backbone fragments served as low affinity competitors for histones in the reconstitution reactions. The mass ratio of array to competitor DNA was 2:1. To determine the histone octamer concentrations needed to saturate the arrays, titrations were performed. Since the mass of a histone octamer (∼108 kDa) is comparable to the mass of 197 bp of DNA (∼122 kDa), the indicated mass ratios of array DNA to histone octamers are similar to the respective molar ratios of Widom-601 nucleosome positioning sequences to octamers. The assembled arrays were purified by MgCl_2_ precipitation [53] with a final magnesium concentration of 3.5 to 4.4 mM for the nucleosome and 3.25 mM for the chromatosome arrays.

#### Quality control of the arrays

Saturation of the arrays with histone octamers and linker histone was controlled essentially as described previously [43]. Array preparations (amounts corresponding to approximately 150–200 ng array DNA) were loaded onto native 0.7% agarose gels in 0.2x TB buffer before and after MgCl_2_ precipitation, and the DNA was visualised by staining with ethidium bromide. Purified arrays (65 fmol according to measurements of the DNA content at 260 nm) were digested into monomers with AvaI (15 U; NEB) in 10 mM Hepes-NaOH pH 7.6, 50 mM KCl, 1.5 mM MgCl_2_, 0.5 mM EGTA pH 8 for 75 min at 26°C (15 µl final volume). The reactions were analysed on 1.1% native agarose gels as above. To probe for accessibility of the AluI site, the arrays (82 fmol) were incubated for 1 h at 26°C with AluI (10 U; NEB) in 25 mM Hepes-KOH pH 7.6, 50 mM NaCl, 1 mM MgCl_2_, 0.1 mM EDTA, 10% glycerol, 1 mM DTT (20 µl final volume). The digest was stopped by addition of EDTA (20–40 mM) and SDS (0.3–1%), followed by Proteinase K treatment (Genaxxon). The array DNA fragments were purified by ethanol precipitation and resolved on ethidium bromide-containing agarose gels. Stoichiometric incorporation of the histone octamers and H1 were controlled by separating the protein content of the purified arrays on SDS gels (15–18%) and staining with Coomassie. The relative protein ratios were determined by quantifying the intensities of the protein bands using the Odyssey Infrared Imaging System (LI-COR). To achieve saturating levels of H1 in the reconstitution reactions, an excess of H1 relative to nucleosomes had to be added. This is presumably due to several factors including presence of the competitor DNA, loss of H1 on plastic surfaces and inaccuracies in protein concentration determination [78].

### Enzyme assays

Unless indicated otherwise, all assays were performed at 26°C in a buffer containing 25 mM Hepes-KOH pH 7.6, 50 mM NaCl, 1 mM MgCl_2_, 0.1 mM EDTA, 0.2 g/l BSA, 10% glycerol and 1 mM DTT or 10 mM β-mercaptoethanol and in presence of an ATP regenerating system consisting of phosphoenolpyruvate (2–6 mM), a pyruvate kinase-lactate dehydrogenase mixture (15.5 U/ml; Sigma) and NADH (0.5 mM). For remodelling and sliding assays, NADH was omitted from the regenerating system. ATP was always added in a stoichiometric complex with Mg^2+^. Concentrations of nucleosome and chromatosome arrays were determined by quantifying the DNA content via absorption measurement at 260 nm. Indicated molar concentrations refer to individual nucleosomes.

#### Steady-state ATPase assay

ATP hydrolysis was measured by a coupled ATPase assay as described [72] with the indicated concentrations and buffer conditions. In the array-stimulated ATPase assay, a 4-fold lower ATP concentration yielded comparable rates within 75% regardless of array type, indicating saturation with ATP. Unmodified and acetylated *D. melanogaster* H4 N-terminal peptides comprising amino acids 1–24 were purchased from Peptide Specialty Laboratories (counter ion: bicarbonate; H4 (and H4K16ac) peptide: TGRGKGGKGLGKGGAK(ac)RHRKVLRD, scrambled peptide: KLRRGGKacGDVKTGKLGGRKAGRGH (ac: acetylation)). The lyophilized peptides were dissolved in 10 mM Hepes-KOH pH 7.6, and relative peptide concentration was controlled by absorption measurement at 214 and 220 nm. Peptide-stimulated ISWI ATPase activity was followed in 25 mM Hepes-KOH pH 7.6, 100 mM KOAc, 1.5 mM Mg(OAc)_2_, 0.1 mM EDTA, 0.2 g/l BSA, 10% glycerol, 1 mM DTT.

#### Remodelling assay

To follow remodelling, arrays were incubated with ATP and ISWI or ACF at the indicated concentrations along with 0.5 U/µl AluI (NEB) [43], [52]. After terminating the reaction by addition of EDTA (20–40 mM) and SDS (0.33–0.4%), the samples were deproteinized. The DNA was purified by ethanol precipitation and resolved on a 0.9% agarose gel. Fluorescently labelled DNA was visualized using the Odyssey Infrared Imaging System (LI-COR), and band intensities were quantified with the Odyssey software. Unlabelled DNA was quantified after ethidium bromide staining by densitometry (AIDA; raytest). To assure that AluI was not limiting, 4–5-fold lower concentrations were tested, yielding results that deviated by less than 2-fold regardless of the reaction conditions. Saturation with ISWI was controlled by using a 3.3-fold lower enzyme concentration. Under these conditions, remodelling was slightly faster (1.6-fold), which is in full agreement with previous observations of ISWI remodelling slowing down with increasing enzyme concentrations [52] and confirmed saturation.

#### Sliding assay

The sliding assay was performed as described [52], [55] with the indicated concentrations. Longer AvaI digests with higher enzyme concentrations yielded comparable results.

## Supporting Information

Figure S1 **Synthesis of histone H4 site-specifically acetylated at lysine 16.** (A) Scheme of the semi-synthetic method applied for generation of the acetylated H4 [32]. A truncated H4 harbouring amino acids (aa) 20–102 with lysine 20 mutated to cysteine (C_20_) was bacterially expressed and purified. Using native chemical ligation, this H4 derivative was N-terminally fused to a chemically synthesized peptide comprising aa 1–19 of H4 carrying an acetylation (Ac) on lysine 16 (K_16_). Next, C_20_ was converted into a lysine analogue (K_S_) by S-alkylation. (B) Structure of lysine (K) and the lysine analogue (K_S_). Except for the thioether in the side chain of the lysine analogue at position 20, the synthesized acetylated H4 bore the canonical aa sequence. (C) Full survey spectrum of the unmodified (top: H4) and the site-specifically acetylated (bottom: H4K16ac) peptide 4–17 of histone H4. The analysis was performed on histones that were incorporated into nucleosome arrays. The protein content of the arrays was separated on an SDS gel, stained with Coomassie, and the histone H4 band was excised. Prior to trypsin digestion, the non-acetylated lysines were chemically acetylated with deuterated acetic anhydride (Ac_3_). Acetylation prevented trypsin from cutting after lysine, and therefore longer peptides were generated. (M: molecule; m/z: mass-to-charge ratio; m: monoisotopic mass value; Δm: difference between the expected and the measured masses; R: resolution of the mass spectrometry measurement). (D) Determination of the acetylated lysine in the monoacetylated peptide H4K16ac. To determine which of the four lysine residues (K5, K8, K12 or K16) within the 4–17 peptide was acetylated, the b- and y-ions were analysed. For the y_5_-ion comprising K16 a peak corresponding to the naturally acetylated ion (+Ac) was detected, whereas no peak corresponding to the chemically acetylated peptide (+Ac_3_) was observed (inset I). Furthermore, for the b_9_-ion comprising the other three lysine residues of the analysed peptide (K5, K8 and K12) only a peak corresponding to the three-times chemically acetylated ion (+3xAc_3_) was detected. No ions carrying one natural acetylation along with two chemically introduced ones were present (+1xAc +2xAc_3_). This result proves that the single natural acetylation in the H4K16ac peptide observed in panel C was indeed located on K16.
(TIF)

Figure S2 **Quality controls of the histone octamers and nucleosome arrays.** (A) Example of a Coomassie-stained SDS gel to control relative histone stoichiometry on purified saturated nucleosome arrays. (B) Native agarose gels of the nucleosome arrays from Figure 1B before and after MgCl_2_ precipitation. Samples of the reconstitution reactions directly after assembly (i), the pellet fraction after MgCl_2_ precipitation (p), and the corresponding supernatant (SN) were loaded. The gels were stained with ethidium bromide after the run. The nucleosome arrays ran well above the 5 kb DNA marker band, where free array DNA would be expected. A homogenous population of fully saturated arrays was indicated by one sharp band. Excess histone octamers present in the reconstitution reaction bound to the competitor DNA resulting in a band shift. After MgCl_2_ precipitation only the nucleosome arrays were retained in the pellet, no contaminating competitor DNA was present. Only fully saturated arrays precipitated quantitatively with MgCl_2_. (C) AluI digests of the purified nucleosome arrays from B. The purified DNA was loaded onto agarose gels and stained with ethidium bromide. In fully saturated arrays (histone octamer to DNA ratio of 1.4:1) all AluI sites were protected by a nucleosome and the array DNA was not cut by the enzyme. Contrary, unoccupied Widom-601 sites in non-saturated arrays exposed an AluI site and got cut, giving rise to a ladder of DNA fragments. (kb: kilobases).
(TIF)

Figure S3 **ISWI ATPase activity in presence of nucleosome and chromatosome arrays.** Result of an exemplary steady-state ATPase assay. The assay was performed as in Figure 2A and 6A employing different concentrations of nucleosome and chromatosome arrays. Reactions were performed in duplicates or triplicates. Data were fit to single exponential functions (dashed lines; Kaleidagraph). Note that no affinities were retrievable, as ISWI at 100 nM was not subsaturating. Nevertheless, nucleosome array concentrations needed for enzyme saturation could be extracted.
(TIF)

Figure S4 **Remodelling of chromatosome arrays reconstituted with different H1 input amounts.** Remodelling of unmodified and acetylated chromatosome arrays assembled with different molar ratios of nucleosomes to H1 (indicated in brackets) was performed and analysed as in Figure 6B, C. Control reactions did not contain ATP.
(TIF)
