# Supplementary figures and images for: ISWI Remodelling of Physiological Chromatin Fibres Acetylated at Lysine 16 of Histone H4
Source: PLoS One. 2014 Feb 6;9(2):e88411. doi: 10.1371/journal.pone.0088411 (PMC3916430; doi:10.1371/journal.pone.0088411)

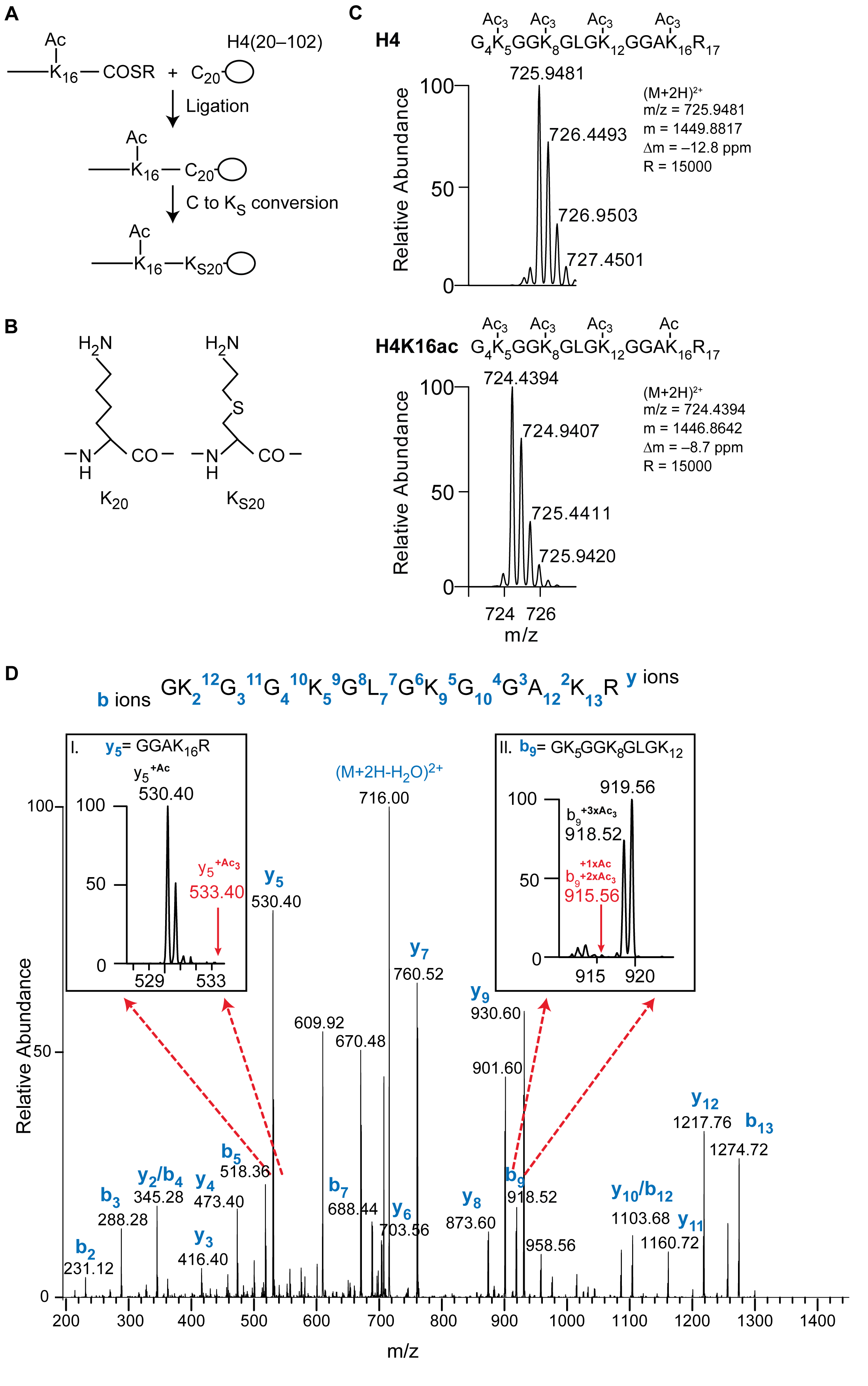

Supplement: Figure S1 — Synthesis of histone H4 site-specifically acetylated at lysine 16. (A) Scheme of the semi-synthetic method applied for generation of the acetylated H4 [32]. A truncated H4 harbouring amino acids (aa) 20–102 with lysine 20 mutated to cysteine (C20) was bacterially expressed and purified. Using native chemical ligation, this H4 derivative was N-terminally fused to a chemically synthesized peptide comprising aa 1–19 of H4 carrying an acetylation (Ac) on lysine 16 (K16). Next, C20 was converted into a lysine analogue (KS) by S-alkylation. (B) Structure of lysine (K) and the lysine analogue (KS). Except for the thioether in the side chain of the lysine analogue at position 20, the synthesized acetylated H4 bore the canonical aa sequence. (C) Full survey spectrum of the unmodified (top: H4) and the site-specifically acetylated (bottom: H4K16ac) peptide 4–17 of histone H4. The analysis was performed on histones that were incorporated into nucleosome arrays. The protein content of the arrays was separated on an SDS gel, stained with Coomassie, and the histone H4 band was excised. Prior to trypsin digestion, the non-acetylated lysines were chemically acetylated with deuterated acetic anhydride (Ac3). Acetylation prevented trypsin from cutting after lysine, and therefore longer peptides were generated. (M: molecule; m/z: mass-to-charge ratio; m: monoisotopic mass value; Δm: difference between the expected and the measured masses; R: resolution of the mass spectrometry measurement). (D) Determination of the acetylated lysine in the monoacetylated peptide H4K16ac. To determine which of the four lysine residues (K5, K8, K12 or K16) within the 4–17 peptide was acetylated, the b- and y-ions were analysed. For the y5-ion comprising K16 a peak corresponding to the naturally acetylated ion (+Ac) was detected, whereas no peak corresponding to the chemically acetylated peptide (+Ac3) was observed (inset I). Furthermore, for the b9-ion comprising the other three lysine residues of the a [file pone.0088411.s001.tif]

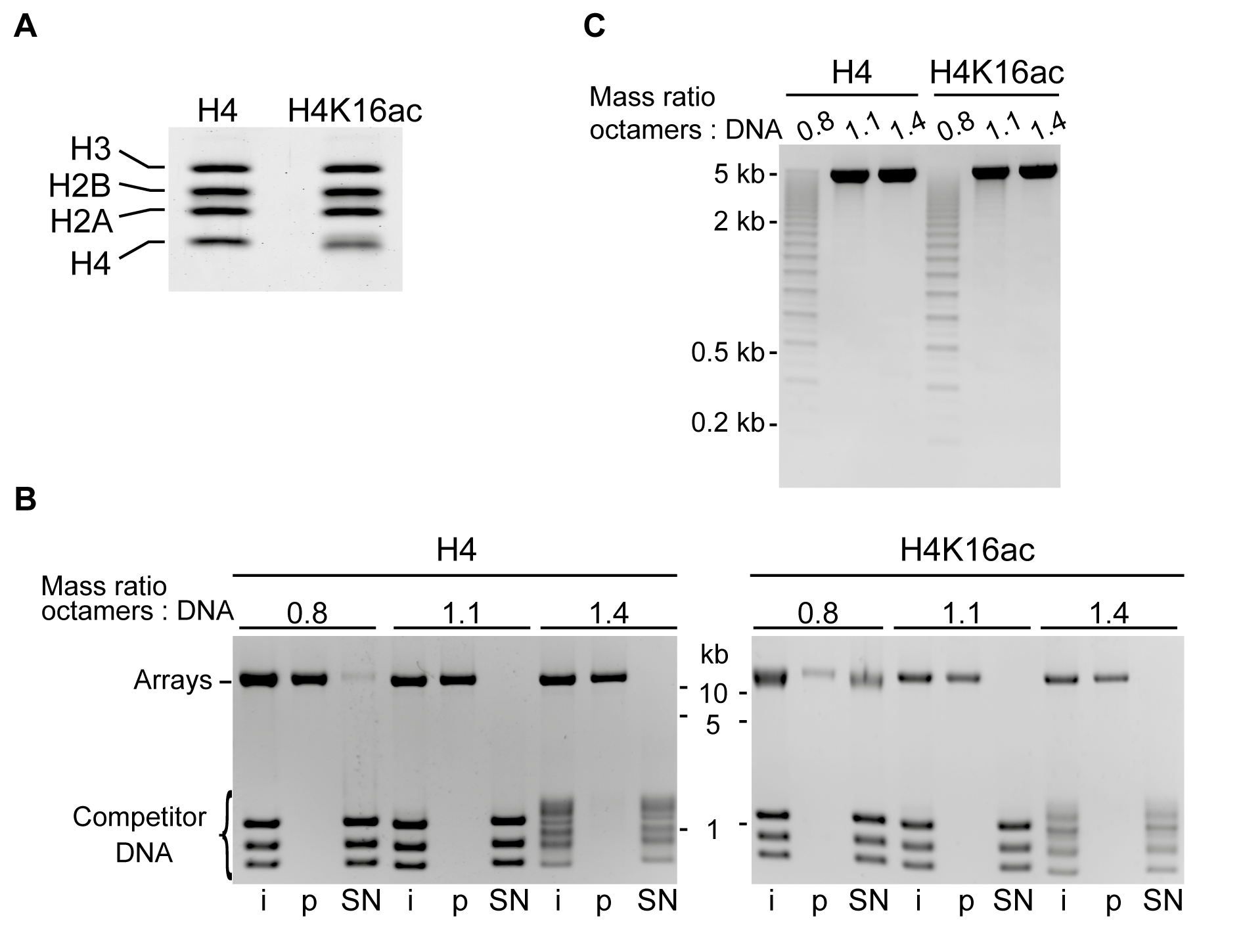

Supplement: Figure S2 — Quality controls of the histone octamers and nucleosome arrays. (A) Example of a Coomassie-stained SDS gel to control relative histone stoichiometry on purified saturated nucleosome arrays. (B) Native agarose gels of the nucleosome arrays from Figure 1B before and after MgCl2 precipitation. Samples of the reconstitution reactions directly after assembly (i), the pellet fraction after MgCl2 precipitation (p), and the corresponding supernatant (SN) were loaded. The gels were stained with ethidium bromide after the run. The nucleosome arrays ran well above the 5 kb DNA marker band, where free array DNA would be expected. A homogenous population of fully saturated arrays was indicated by one sharp band. Excess histone octamers present in the reconstitution reaction bound to the competitor DNA resulting in a band shift. After MgCl2 precipitation only the nucleosome arrays were retained in the pellet, no contaminating competitor DNA was present. Only fully saturated arrays precipitated quantitatively with MgCl2. (C) AluI digests of the purified nucleosome arrays from B. The purified DNA was loaded onto agarose gels and stained with ethidium bromide. In fully saturated arrays (histone octamer to DNA ratio of 1.4:1) all AluI sites were protected by a nucleosome and the array DNA was not cut by the enzyme. Contrary, unoccupied Widom-601 sites in non-saturated arrays exposed an AluI site and got cut, giving rise to a ladder of DNA fragments. (kb: kilobases). (TIF) [file pone.0088411.s002.tif]

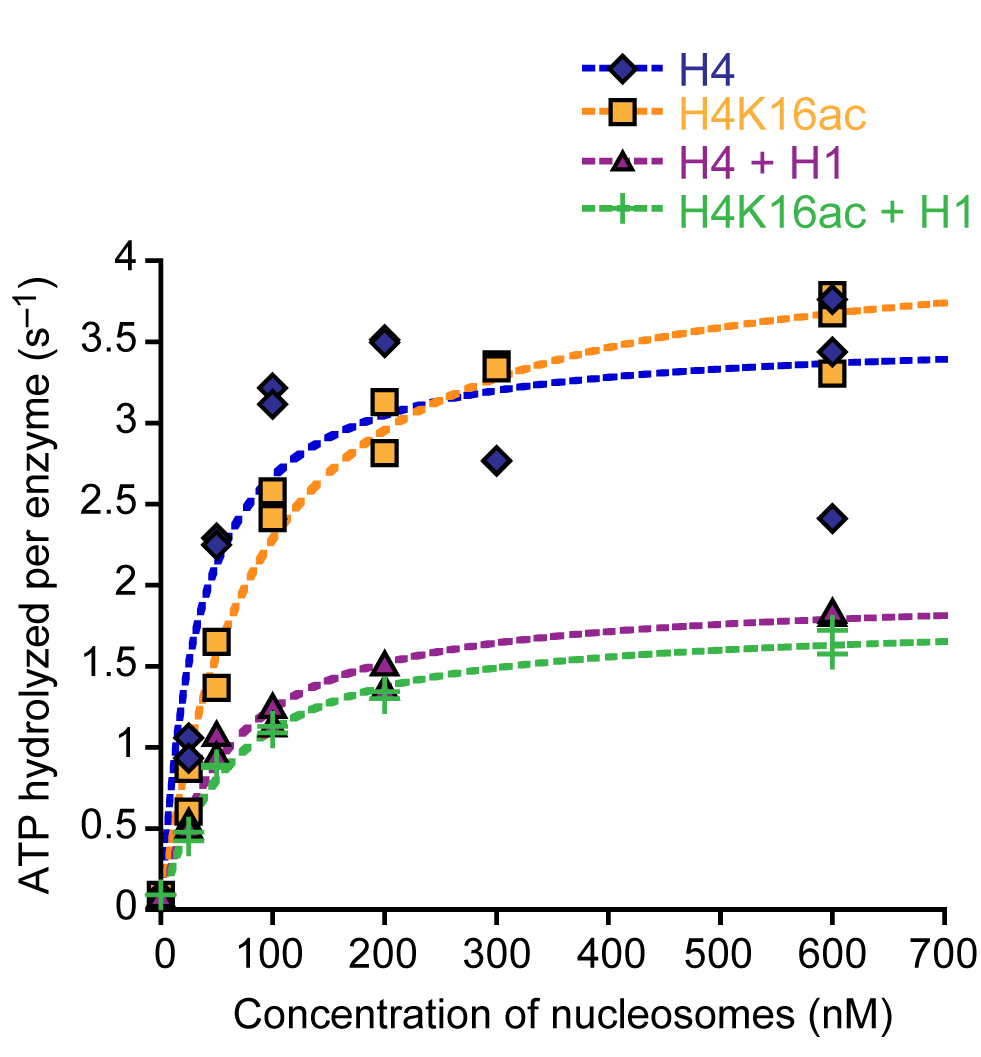

Supplement: Figure S3 — ISWI ATPase activity in presence of nucleosome and chromatosome arrays. Result of an exemplary steady-state ATPase assay. The assay was performed as in Figure 2A and 6A employing different concentrations of nucleosome and chromatosome arrays. Reactions were performed in duplicates or triplicates. Data were fit to single exponential functions (dashed lines; Kaleidagraph). Note that no affinities were retrievable, as ISWI at 100 nM was not subsaturating. Nevertheless, nucleosome array concentrations needed for enzyme saturation could be extracted. (TIF) [file pone.0088411.s003.tif]

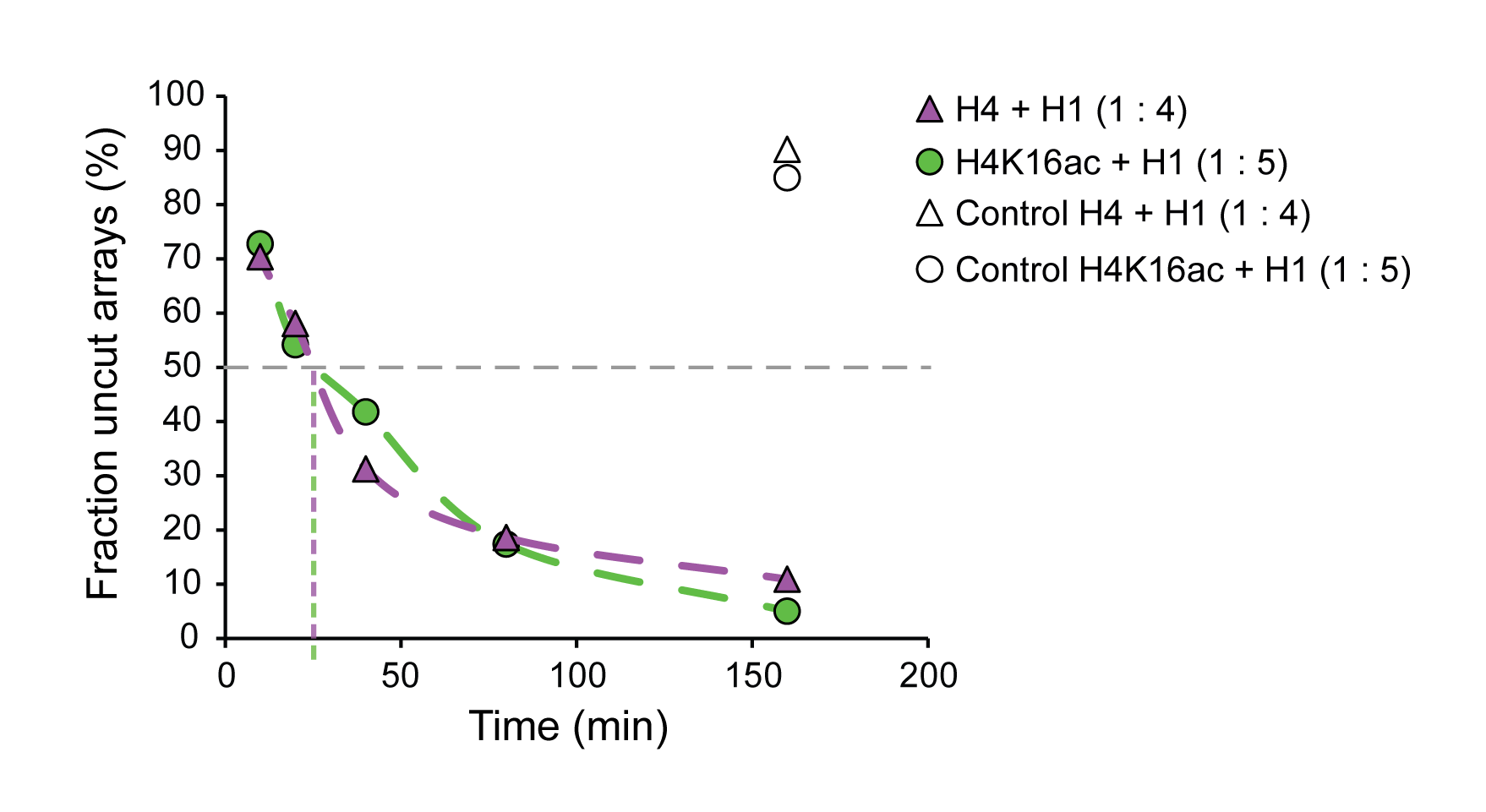

Supplement: Figure S4 — Remodelling of chromatosome arrays reconstituted with different H1 input amounts. Remodelling of unmodified and acetylated chromatosome arrays assembled with different molar ratios of nucleosomes to H1 (indicated in brackets) was performed and analysed as in Figure 6B, C. Control reactions did not contain ATP. (TIF) [file pone.0088411.s004.tif]
